# Transbuccal With Transoral Versus Transoral-Only Approach for Open Reduction and Internal Fixation of Mandibular Angle Fractures: A Prospective Study

**DOI:** 10.7759/cureus.90784

**Published:** 2025-08-22

**Authors:** Ali Qamar, Nandakishore Donepudi, Deepak Thakur, Akansh Datta, Anshi Jain, Anushka Singh, Saayam Jain, Kriti Agarwal

**Affiliations:** 1 Department of Oral and Maxillofacial Surgery, Teerthanker Mahaveer Dental College and Research Centre, Moradabad, IND

**Keywords:** mandibular fractures, maxillofacial surgery, open reduction, transbuccal, transoral

## Abstract

Introduction: Mandibular angle fractures, a common type of maxillofacial trauma, often require surgical intervention through open reduction and internal fixation (ORIF) due to the intricate interplay of muscle forces causing displacement. This study compared the transbuccal combined with transoral approach (transbuccal with transoral approach) and the standalone transoral approaches for ORIF of mandibular angle fractures, evaluating ease of surgical access, fracture fixation stability, surgical duration, radiographic alignment, facial scarring, and postoperative occlusion to determine the optimal method.

Materials and Methods: This prospective, non-randomized clinical study enrolled 32 patients (aged 18-60 years) with mandibular angle fractures at Teerthanker Mahaveer Dental College and Research Centre, Moradabad, India, over 18 months. Patients were allocated to Group A (transbuccal with transoral approach, n = 16, using 3D miniplates) or Group B (standalone transoral approach, n = 16, using 4-hole miniplates) based on the surgeon's preference. Outcomes were assessed by calibrated surgeons: ease of access (good, fair, poor), stability (bimanual palpation on Day 2 and 3 months), surgical duration (incision to closure), radiographic alignment on Day 2, scarring (hypertrophic, barely visible, invisible at 1 and 3 months), and occlusion (satisfactory, mild discrepancy, unsatisfactory at 5 and 10 days). Statistical analysis used independent t-tests and chi-square tests (p < 0.05).

Results: Group A showed complete male predominance (p = 0.032), with no age difference (Group A: 27.12 ± 6.45 years; Group B: 29.37 ± 8.34 years; p = 0.36). Both groups achieved 100% stability on Day 2 and at 3 months (p = 1). Group A had longer surgical times (101.56 ± 7.33 min vs. 69.62 ± 11.89 min; p=0.001). No differences were found in ease of access (p = 0.344) or alignment (p = 0.867). Group A exhibited 100% mild scarring at 1 month and 75% invisible scars at 3 months, while Group B had no visible scars. Occlusion outcomes favored Group A at 5 and 10 days (p < 0.05).

Conclusion: Both approaches ensured excellent stability and alignment, but the transbuccal with transoral approach offered superior occlusal outcomes and minimal scarring, despite longer surgical times, making it preferable for complex fractures. The transoral approach, with shorter duration and no external scarring, is suitable for patients who prioritize aesthetics.

## Introduction

Mandibular fractures, particularly those involving the angle, are a significant subset of maxillofacial injuries, accounting for 20-30% of such cases due to the mandible’s prominent anatomical position and susceptibility to trauma [1]. The complexity of mandibular angle fractures arises from their unique biomechanical environment, influenced by the opposing forces of the masticatory and suprahyoid muscles, which often result in fracture displacement [2]. This often requires open reduction and internal fixation (ORIF) to restore anatomical alignment, ensure functional recovery, and minimize complications such as infection, malocclusion, or non-union, which have a reported incidence of 0-32% in angle fractures [3]. Effective management is critical not only for functional outcomes like mastication and speech but also for aesthetic considerations and patient safety, given the potential for airway compromise and bacterial contamination from the oral cavity [4].

The evolution of ORIF techniques has significantly improved outcomes for mandibular angle fractures [3,4]. Surgical approaches for ORIF of mandibular angle fractures include the transoral approach and the transbuccal with transoral approach [3,5]. The transoral approach, performed through an incision in the oral mucosa or buccal vestibule, avoids external scarring and facial nerve damage, making it a preferred method [3]. However, it poses challenges such as suboptimal plate positioning, limited soft tissue coverage, increasing risks of wound dehiscence and hardware exposure, and higher chances of screw loosening due to reduced bone density in the upper mandible [6]. The transbuccal with transoral approach, involving a transoral incision combined with a small facial stab incision for trocar placement, addresses these limitations by enabling plate fixation on the thicker lateral mandibular surface, reducing plate bending, and improving soft tissue coverage [5]. This approach also minimizes infection risk from third molar regions and allows direct visualization of occlusion during fixation, potentially enhancing outcomes.

The choice of surgical approach depends on factors such as fracture visibility, displacement extent, accessibility, surgeon expertise, and patient preferences. While the transoral approach prioritizes aesthetics by avoiding external scars, the transbuccal with transoral approach offers technical advantages in stability and reduced complications [3,5-7]. This study aimed to compare the transbuccal with transoral approach versus the standalone transoral approach for ORIF of mandibular angle fractures by evaluating ease of surgical access, stability of fracture fixation, surgical duration from incision to closure, radiographic accuracy of mandibular reduction, aesthetic outcomes related to facial scarring, and postoperative occlusal alignment.

## Materials and methods

This prospective, comparative clinical study was conducted at the Department of Oral and Maxillofacial Surgery, Teerthanker Mahaveer Dental College and Research Centre, Moradabad, India. The study spanned over a period of 18 months, adhering to the ethical principles outlined in the Declaration of Helsinki. Ethical clearance was obtained from the Institutional Ethical Committee (TMDCRC/IEC/TH/22-23/OMFS 01). Informed consent was secured from all participants, with detailed explanations of the procedure, potential risks, and follow-up requirements provided in written and verbal formats, ensuring voluntary participation and the right to withdraw at any time without consequence.

Patients aged 18-60 years with unilateral or bilateral mandibular angle fractures, classified as ASA I or ASA II (including those with controlled systemic conditions like diabetes or hypertension within normal ranges) according to the American Society of Anesthesiologists (ASA) [8], and willing to comply with follow-up visits were included. Exclusion criteria encompassed mentally challenged or medically compromised patients (such as uncontrolled systemic diseases), those in the first or third trimester of pregnancy, and individuals unable to provide consent or attend follow-ups.

The required sample size was determined using G*Power software version 3.1.9.2 (Heinrich Heine University, Düsseldorf, Germany). Based on an effect size of 0.9 (derived from a prior study comparing surgical time and fracture gap between transoral and transbuccal approaches for mandibular angle fractures), a minimum of 32 participants (16 per group) was found to be sufficient [9]. This calculation ensured 80% statistical power with a 95% confidence level for the present study.

Participants were allocated to two groups based on the surgeon’s clinical judgment and preference, considering factors such as fracture displacement, accessibility, and anatomical considerations, without randomization to reflect real-world clinical decision-making. Group A underwent ORIF using the transbuccal with transoral approach with a three-dimensional (3D) miniplate (Ortho Max, Mumbai, India), while Group B received ORIF via the transoral approach alone using a four-hole miniplate (Ortho Max, Mumbai, India). To ensure standardization, all procedures were performed by experienced oral and maxillofacial surgeons trained in both techniques, with calibration achieved through preoperative workshops to standardize incision techniques, plate placement, and outcome assessment protocols. Surgical instruments, including retractors, drills, and screwdrivers, were sourced from Ortho Max (Mumbai, India) and standardized across procedures to minimize variability.

The surgical protocol began with patient admission, followed by a detailed case history, routine blood tests, and radiographic investigations (panoramic radiograph or cone-beam computed tomography, or CBCT). Pre-anesthetic clearance was obtained, and preoperative medications were administered 1 h prior to surgery: ceftriaxone 1 gm IV (Ceftum, GlaxoSmithKline, Brentford, UK) and dexamethasone 8 mg IV (Dexona, Zydus Cadila, Ahmedabad, India). On the operating table, patients were scrubbed and draped with povidone-iodine solution (Betadine, Win-Medicare, New Delhi, India). Intermaxillary fixation was achieved using stainless steel wires (Ortho Max, Mumbai, India) to stabilize occlusion. For both groups, a transoral vestibular incision (5-7 mm) was made from the premolar region to the third molar area, deepened subperiosteally, and a full-thickness mucoperiosteal flap was raised. The fracture site was debrided, anatomically reduced, and fixed with miniplates.

**Figure 1:**
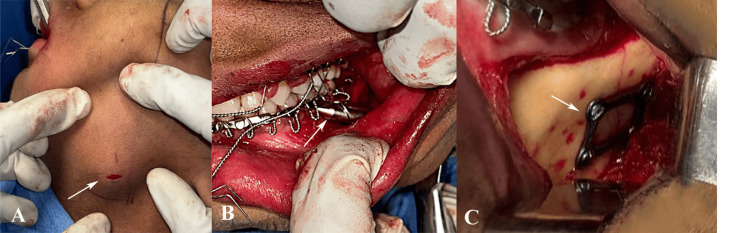
Transbuccal with transoral approach for open reduction and internal fixation (ORIF) of mandibular angle fracture. (A) Initial extraoral exposure of the fracture site (B) Placement of transbuccal trocar for lateral access, (C) Fixation of the fracture using three-dimensional miniplates and screws through the combined approach. Original clinical images of a patient from the study, used with the patient’s informed consent.

In Group A, a transbuccal trocar was placed within a safety zone defined by three lines: the trago-basal line (from the tragus to the antero-inferior masseter groove), the cantho-gonial line (from the outer canthus to the mandibular angle), and the mandibular border. This facilitated precise plate placement on the lateral mandibular surface (Figure 1).

**Figure 2:**
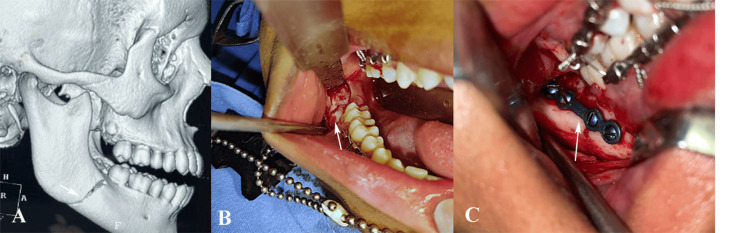
Transoral approach for open reduction and internal fixation (ORIF) of mandibular angle fracture, (A) Cone-beam computed tomographic scan showing unilateral mandibular angle fracture, (B) Intraoral exposure of the mandibular angle fracture site, (C) Fixation of the fracture using 4-hole miniplates and screws via intraoral access. Original clinical images of a patient from the study, used with the patient’s informed consent.

In Group B, the transoral approach alone was used, with the 4-hole miniplate positioned along the upper border. Closure was performed in layers using Vicryl resorbable sutures (Ethicon, Johnson & Johnson, New Brunswick, NJ, USA) for deeper layers and silk sutures (Ethicon, Johnson & Johnson, New Brunswick, NJ, USA) for mucosal closure after achieving hemostasis (Figure 2).

Postoperative care followed a standardized regimen for 48-72 h: amoxicillin + clavulanic acid 1.2 gm IV twice daily (Augmentin, GlaxoSmithKline, Brentford, UK), metronidazole 500 mg/100 ml IV three times daily (Metrogyl, J.B. Chemicals, Mumbai, India), diclofenac sodium 75 mg IM twice daily (Voveran, Novartis, Basel, Switzerland), dexamethasone 8 mg IV once daily, ranitidine 300 mg IV once daily (Rantac, J.B. Chemicals, Mumbai, India), ondansetron 4 mg IV as needed (Emeset, Cipla, Mumbai, India), and fluids (DNS 500 ml and RL 500 ml, Baxter, Deerfield, IL, USA).

Upon discharge, patients received oral medications for five days: amoxicillin 500 mg + clavulanic acid 125 mg twice daily (Augmentin, GlaxoSmithKline, Brentford, UK), metronidazole 400 mg three times daily (Metrogyl, J.B. Chemicals, Mumbai, India), aceclofenac 100 mg + paracetamol 325 mg + serratiopeptidase 15 mg twice daily (Zerodol-SP, Ipca Laboratories, Mumbai, India), pantoprazole 40 mg once daily (Pantocid, Sun Pharma, Mumbai, India), vitamin B complex + vitamin C capsules once daily (Becosules-Z, Pfizer, New York, NY, USA), and chlorhexidine 0.12% mouthwash 3-4 times daily (Hexidine, Icpa Health Products, Ankleshwar, India). Patients were instructed to maintain a soft diet and oral hygiene.

**Figure 3:**
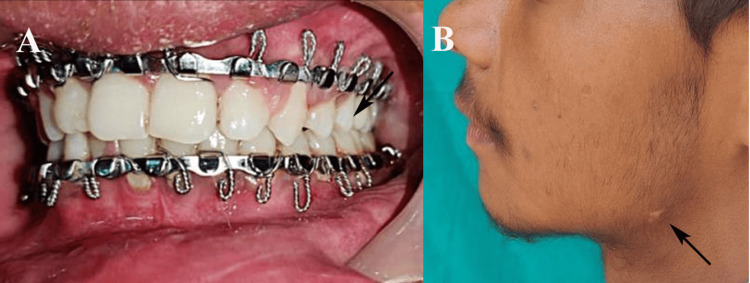
(A) Postoperative occlusion assessment, (B) postoperative assessment of facial scar in transbuccal with transoral approach at the one-month follow-up. Original clinical images of a patient from the study, used with the patient’s informed consent.

Outcome assessments were conducted by the operating surgeon, with calibration ensured through intra-rater reliability training. Ease of surgical access was graded as good, fair, or poor, based on intraoperative maneuverability and visibility [10]. Stability of fracture fixation, assessed via bimanual palpation on the second postoperative day and at three months, was classified as stable (no appreciable mobility) or unstable (clinically detectable mobility) [1]. Surgical duration was measured using a stopwatch (Casio, Tokyo, Japan) from incision to the final suture placement. Radiographic assessment of mandibular reduction was performed using a panoramic radiograph or CBCT on the second postoperative day to evaluate alignment accuracy. Facial scarring was assessed at one and three months as hypertrophic, invisible, or barely visible for transbuccal with transoral approach [10]. Postoperative occlusion was evaluated on the fifth and tenth days as satisfactory, mild discrepancy (<2 mm), or unsatisfactory (>2 mm) (Figure 3).

The data were analyzed using Statistical Package for Social Sciences (SPSS) software version 20 (IBM Corp., Armonk, NY, USA). Categorical variables were presented as frequencies and percentages, while continuous variables such as age and surgical time were expressed as mean ± standard deviation. For group comparisons, categorical variables were analyzed using either the chi-square test or Fisher's exact test (when expected cell counts were less than 5), and continuous variables were compared using independent samples t-tests. A p-value of less than 0.05 was considered statistically significant for all analyses.

## Results

The comparative analysis between groups revealed several significant findings. Sex distribution showed male predominance in Group A compared to Group B (p = 0.032). Both groups demonstrated 100% stability at Day 2, while three-month stability showed no significant difference (p = 1). Significant differences emerged in scar formation, with Group A showing 100% mild scarring at one month and 75% invisible scars at three months. Occlusion outcomes significantly favored the transbuccal with transoral approach, with better results at both five days and 10 days. Though no significant differences were noted in ease of access (p = 0.344); however, the transbuccal with transoral approach provided good to fair access in most of the patients, which is a definitive advantage over the transoral-only approach. These results suggest that while both techniques provide excellent early stability, the transbuccal with transoral approach offers advantages in terms of occlusion outcomes. The complete absence of visible facial scarring in the transoral group represents a potential cosmetic advantage that warrants consideration (Table 1).

**Table 1:** Comparison of categorical variables between study groups. Chi-square test was applied for all variables, whereas, Fisher exact test was used to assess stability at third month post-treatment and occlusion at tenth day post-treatment, *p < 0.05 denotes statistical significance. Group A used transbuccal with transoral approach, Group B used transoral approach alone. *p < 0.05 denotes statistical significance, Data are presented as frequency (N) and percentage (%), where N denotes number of participants.

Variables	Categories	Group A		Group B		Test statistics	p-value
		N	%	N	%		
Sex	Male	16	100	12	75	4.57	0.032*
	Female	0	0	4	25		
Ease of access	Good	10	63	6	38	2.13	0.344
	Fair	5	31	8	50		
	Poor	1	6	2	13		
Stability on the second day post-treatment	Yes	16	100	16	100	Not applicable	Not applicable
	No	0	0	0	0		
Stability at three-month post-treatment	Yes	15	94	14	88	0	1
	No	1	6	2	13		
Lower border of the mandible	Approximate	9	56	8	50	0.28	0.867
	Gap <3mm	6	38	6	38		
	Gap >3mm	1	6	2	13		
Scarring at one month post-treatment	Invisible	0	0	0	0	Not applicable	Not applicable
	Mildly visible	16	100	0	0		
	Hypertrophic	0	0	0	0		
Scarring at three months post-treatment	Invisible	12	75	0	0	Not applicable	Not applicable
	Mildly visible	4	25	0	0		
	Hypertrophic	0	0	0	0		
Occlusion on the fifth day post-treatment	Satisfied	14	88	6	38	9.71	0.007*
	Mild discrepancy	2	13	8	50		
	Moderate discrepancy	0	0	2	13		
Occlusion on the 10th day post-treatment	Satisfied	16	100	12	75	5.65	0.022*
	Mild discrepancy	0	0	2	13		
	Moderate discrepancy	0	0	2	13		

The mean age was comparable between Group A (27.12 ± 6.45 years) and Group B (29.37 ± 8.34 years), with no significant difference (t = -0.93, p = 0.36). However, Group A had significantly longer mean surgery time (101.56 ± 7.33 min) compared to Group B (69.62 ± 11.89 min) (t = 9.96, p = 0.001), indicating a statistically significant difference in operative duration between the two groups (Table 2).

**Table 2:** . Comparison of groups for continuous variables. *p < 0.05 denotes statistical significance using independent t-test, Data are presented as mean and standard deviation (SD). Goup A used transbuccal with transoral approach, Group B used transoral approach alone.

Variables	Group A		Group B		Test statistics	p-value
	Mean	SD	Mean	SD		
Age (years)	27.12	6.45	29.37	8.34	-0.93	0.36
Surgery time (minutes)	101.56	7.33	69.62	11.89	9.96	0.001*

## Discussion

The results of the study provided valuable insights into the comparative efficacy of these approaches, revealing both similarities and distinct advantages that align with or diverge from existing literature. The findings highlight the transbuccal/transoral approach’s superior occlusal outcomes, ease of access, and minimal scarring, despite longer surgical times, and offer implications for clinical decision-making in maxillofacial surgery. A meta-analysis conducted by Al-Moraissi and Ellis [11] concluded that the transbuccally placed lateral miniplate was better at reducing the incidence of postoperative complications than one placed on the external oblique ridge using a transoral approach. 

The study noted a male predominance in the transbuccal with transoral approach group compared to the transoral approach alone group. This finding may reflect selection bias due to the non-randomized design, where female patients preferred the transoral-only approach as they did not want any facial scarring. Moreover, in the transbuccal with transoral approach, male patients, due to their anatomical considerations, such as thicker soft tissue or larger mandibular dimensions, facilitate trocar placement [12]. Both groups demonstrated 100% stability on the second postoperative day, with no significant difference at three months. This finding supports the efficacy of both approaches in achieving initial and long-term fracture stability, consistent with Champy et al.’s [13] biomechanical principles, which advocate for miniplate fixation along the mandibular upper border to counteract tensile forces. The use of 3D miniplates in the transbuccal with transoral approach and four-hole miniplates in the transoral approach likely contributed to this outcome, as both systems are designed to provide rigid fixation. A previous study by Danda [14] reported that single non-compression miniplates for treating non-comminuted fractures of the mandibular angle provided similar results as two plates. Our results suggest that modern mini-plate systems, combined with meticulous surgical technique, mitigate these risks effectively.

A significant difference was observed in surgical duration, with the transbuccal and transoral approach requiring longer than the transoral approach. This aligns with the technical complexity of the transbuccal approach, which involves additional steps such as trocar placement and external stab incision. Sugar et al. [15] reported similar findings, noting that the transbuccal with transoral approach takes a longer time compared to the transoral approach alone due to the need for precise identification of the safety zone and trocar manipulation. Sehrawat et al. [9] reported similar findings, where they reported less surgical time with the transoral approach. In contrast, Khandeparker et al. [10] reported that both transbuccal and transoral approaches took a similar surgical time of 37 min.

No significant difference was found in the radiographic assessment of mandibular reduction, indicating that both approaches achieve comparable anatomical alignment. This is consistent with a review by Ellis [3], which reported that both transoral and transbuccal approaches, when performed with intermaxillary fixation and mini-plate systems, yield excellent reduction outcomes on postoperative OPG or CBCT. The use of standardized surgical protocols and intermaxillary fixation in our study likely contributed to this equivalence. However, our findings contrast with an earlier study by Khandeparker et al. [10], who reported better reduction with the transbuccal approach compared to the transoral approach. They reported that the advantageous placement of the miniplate within the transbuccal technique might have facilitated enhanced regulation of both tensile and compressive forces, leading to a more consistent reduction in the fracture gap. Advances in imaging and surgical training likely account for the improved outcomes observed in our study, highlighting the importance of modern technology in achieving precise reduction.

The transbuccal with transoral approach demonstrated 100% mild scarring at one month and 75% invisible scars at three months, while the transoral approach alone showed no visible facial scarring, which is a definitive advantage of the transoral-only approach. The transbuccal approach’s small stab incision, placed within the safety zone defined by trago-basal, cantho-gonial, and mandibular lines, minimizes aesthetic impact, as supported by Sugar et al. [15], who noted that transbuccal incisions heal with minimal scarring when properly placed. The transoral approach’s complete absence of external scars aligns with its cosmetic advantage, as emphasized by Al-Moraissi and Ellis [11], who reported patient preference for transoral techniques due to no visible scarring. However, the mild scarring in the transbuccal approach at one month, transitioning to mostly invisible scars by three months, suggests that the transbuccal approach’s aesthetic impact is temporary and acceptable, especially in male patients with thicker skin.

Occlusion outcomes significantly favored the transbuccal with transoral approach at both 5 and 10 days, with better results than the transoral approach alone. This is likely due to the transbuccal approach’s direct visualization of the fracture site and occlusion during fixation, as highlighted by Khandeparker et al. [10]. The ability to confirm occlusal alignment intraoperatively reduces postoperative discrepancies, a key advantage over the transoral approach, where limited visibility may complicate precise reduction. These findings align with Sugar et al. [15], who reported superior occlusal outcomes with transbuccal approaches due to enhanced access to the lateral mandibular surface. In contrast, Ellis [3] found no significant occlusal differences between approaches when intermaxillary fixation was used, suggesting that our study’s results may reflect surgeon expertise or specific fracture patterns. The transbuccal approach’s advantage in occlusion underscores its utility in complex cases requiring precise occlusal restoration. Wan et al. [16] conducted a study to compare the transbuccal approach and transoral approaches for the management of mandibular angle fractures. The authors included 597 patients and concluded that the transbuccal approach showed fewer complications, such as wound dehiscence, malunion or non-union, and infections, than the transoral approach when used for the treatment of mandibular angle fractures. 

Clinical implications

The findings suggest that the transbuccal with transoral approach offers advantages in occlusal outcomes and acceptable scarring, making it a viable option for mandibular angle fractures, particularly in male patients or those with complex fractures requiring precise alignment. The transoral approach, with its shorter surgical time and absence of external scarring, remains preferable for patients who prioritize aesthetics or in cases with straightforward fractures. Surgeons should weigh these factors against fracture characteristics, patient preferences, and their own expertise. The use of 3D miniplates in the transbuccal approach may enhance stability in biomechanically challenging fractures, while the transoral approach’s simplicity supports its use in resource-limited settings. Both approaches benefit from modern imaging and fixation systems, ensuring high stability and alignment.

Limitations

The non-randomized design introduces potential selection bias, as surgeon preference influenced group allocation, possibly contributing to the male predominance in the transbuccal approach group. The small sample size (n = 32) limits statistical power, particularly for scarring outcomes, which could not be fully analyzed. The study’s three-month follow-up may not capture long-term complications such as plate failure or delayed non-union. Variability in surgeon experience and fracture complexity could also affect outcomes, despite calibration efforts. 

## Conclusions

The study concluded that both techniques achieved excellent early and long-term fracture stability and comparable mandibular alignment. The transbuccal with transoral approach demonstrated superior postoperative occlusal outcomes and minimal scarring, despite requiring longer surgical time, making it a preferred option for cases prioritizing precise occlusion and acceptable aesthetics. The transoral approach, with its shorter operative duration and absence of external scarring, remained advantageous for patients valuing cosmetic outcomes or simpler fractures. These findings support tailored surgical decision-making based on fracture complexity, patient preferences, and surgeon expertise.
